# The Association between Sexual Orientation and Sleep Problems: Are there Racial and Ethnic Differences?

**DOI:** 10.3390/clockssleep1020019

**Published:** 2019-04-23

**Authors:** Elbert P. Almazan

**Affiliations:** Sociology Program, Department of Sociology, Anthropology, and Social Work, College of Liberal Arts and Social Sciences, Central Michigan University, Mount Pleasant, MI 48859, USA; almaz1ep@cmich.edu

**Keywords:** sexual orientation, sexual minority, heterosexual, Asian, Pacific Islander, Black, Latinx, White, sleep, race, ethnicity

## Abstract

Using the 2013–2017 National Health Interview Survey, this study examined whether there were significant sexual orientation differences in sleep problems in specific racial and ethnic populations. The analysis had a large sample size and enough statistical power to evaluate any sexual orientation differences or non-differences in sleep problems in Latinx, non-Latinx Black, non-Latinx Asian and Pacific Islander, and non-Latinx White populations. Consistent with recent studies on sexual orientation and sleep problems, this analysis revealed that, for most racial and ethnic groups, there was no significant sexual orientation difference in non-normal sleep duration. Sexual minority adults were significantly more likely to report not feeling rested, trouble falling asleep, trouble staying asleep, and taking medications for sleep than heterosexual adults. There were two notable exceptions in the findings. The first exception was that Latinx sexual minority adults were significantly more likely to report non-normal sleep duration when compared with Latinx heterosexual adults. The second exception was that there was no significant sexual orientation difference in not feeling rested among Asian and Pacific Islander adults.

## 1. Introduction

This study examined whether there were racial and ethnic differences in the association between sexual orientation and sleep problems. This research builds on recent studies on sexual orientation and sleep problems that analyzed data from the National Health Interview Survey (NHIS) [1,2]. The NHIS provides a nationally representative sample of the sexual minority population in the United States; however, the NHIS sample may be too small to distinguish whether a lack of a significant sexual orientation difference in health is due to low statistical power or an actual sexual orientation non-difference in the population.

In a study using the 2013–2014 NHIS by Chen and Shiu [1], sexual minority adults were more likely to report feeling not rested, trouble falling asleep, and trouble staying asleep than heterosexual adults. They found no sexual orientation difference in non-normal sleep duration. They found that the association between sexual orientation and sleep problems were not different between White adults and non-White adults. A study using the 2013–2015 NHIS by Galinsky et al. [2] builds on Chen and Shiu’s study with a larger sexual minority sample and greater statistical power. This study also found that sexual minority adults were more likely to report feeling not rested, trouble falling asleep, and trouble staying asleep. In addition, they found that sexual minority adults were more likely to report taking medications for sleep than heterosexual adults. They also found no sexual orientation difference in non-normal sleep duration.

The current study used the 2013–2017 NHIS to examine the association between sexual orientation and sleep problems within Latinx, non-Latinx Black, non-Latinx Asian and Pacific Islander, and non-Latinx White populations. Using five years of NHIS data, this large sample size of sexual minority adults who are racial and ethnic minority adults has enough statistical power to evaluate for any sexual orientation difference or non-difference in non-normal sleep duration or reports of feeling not rested, trouble falling asleep, trouble staying asleep, and taking medications for sleep. Studies on the associations between race or ethnicity and sleep problems suggest that the association between sexual orientation and sleep problems may differ according to specific racial or ethnic populations. In a national 2010 study from the National Sleep Foundation [3], Black, Latinx, and White adults were more likely to report having experienced a good night’s sleep up to a few nights a month when compared with Asian adults. Latinx, Black, and White adults were more likely to report taking medications for sleep than Asian adults. Black adults were more likely to report non-normal sleep duration than Latinx, Asian, and White adults. In a study that analyzed survey data from the 2014 Behavioral Risk Factor Surveillance System, Black adults were more likely to report non-normal sleep duration than Latinx, Asian, and White adults [4].

## 2. Data and Methods

### 2.1. Data

The National Health Interview Survey is a publicly available cross-sectional health survey of the U.S. civilian, non-institutionalized population produced by the National Center for Health Statistics (NCHS) of the Centers for Disease Control and Prevention [5]. The NHIS has included a survey question regarding sexual orientation identity for adult participants since 2013. This study used a sample of approximately 165,000 adults who were asked the sexual orientation identity question in the five survey years between 2013 and 2017.

### 2.2. Variables

Women who chose the “lesbian or gay” or “bisexual” categories and men who chose the “gay” or “bisexual” categories in the sexual orientation identity question were classified as “sexual minority” adults. “Sexual minority” and “heterosexual” were coded as dummy variables. A dummy variable of missing sexual orientation information was also created to act as a control in the analysis. 

Race and ethnicity variables were coded as dummy variables. The dummy race and ethnicity variables were “Latinx”, “Non-Latinx Black”, “Non-Latinx Asian and Pacific Islander”, and “Non-Latinx White.” A dummy variable of “Other Race and Ethnicity” was also created to be a control in the analysis.

The sleep problem variables followed the coding from the Galinsky et al. study, which used recommendations from the National Sleep Foundation [2]. “Non-Normal Sleep Duration” was a dummy variable in which the survey respondent did not report seven to nine hours as the average number of hours of sleep per day if they were between the age of 18 to 64 or if they did not report seven to eight hours if they were older than 65. “Not Feeling Well Rested” was a dummy variable in which the survey respondent reported not feeling rested for four or more days in the past week. “Trouble Falling Asleep” was a dummy variable in which the survey respondent reported having trouble falling asleep four or more times in the past week. “Trouble Staying Asleep” was a dummy variable in which the survey respondent reported having trouble staying asleep four or more times in the past week. “Taking Medications For Sleep” was a dummy variable in which the survey respondent reported taking medication to help go to sleep four or more times in the past week. 

Control variables in the analysis included variables on gender, age, education level, and family poverty level. Multiple imputations were conducted under procedures recommended by NCHS for missing data on the family poverty level variables [6]. Because of missing information on sleep problems and education, 4.3% of the sample was dropped. After missing data were dropped, the sample size was 157,562.

### 2.3. Statistical Analysis

All statistical estimates used weights. In some statistical analyses, some survey participants were dropped because some strata did not have the specific racial or ethnic minority adults of interest. Sexual minority was the main independent variable of interest. Heterosexual was the reference category for the sexual minority variable. The sleep problems variables were the dependent variables, and logistic regression was conducted. Regression equations were estimated separately for Latinx, non-Latinx Black, non-Latinx Asian and Pacific Islander, and non-Latinx White populations. Regression equations controlled for missing sexual orientation information, gender, age, education level, and family poverty level. Odds ratios and standard errors were evaluated for any significance sexual orientation differences in sleep problems in each racial and ethnic population.

## 3. Results

Table 1 displays the weighted descriptive statistics of sexual orientation identity, race and ethnicity, and sleep problems. Sexual minority adults constituted 2.7% of the sample. Heterosexual adults constituted 95.9% of the sample. Latinx adults constituted 11.1% of the sample. Non-Latinx Black adults constituted 12.3% of the sample. Non-Latinx Asian and Pacific Islander adults constituted 4.9% of the sample. Non-Latinx White adults constituted 68.6% of the sample. “Non-Normal Sleep Duration” constituted 37.5% of the sample. “Not Feeling Well Rested” constituted 36.2% of the sample. “Trouble Falling Asleep” constituted 15.7% of the sample. “Trouble Staying Asleep” constituted 22.2% of the sample. “Taking Medications For Sleep” constituted 8.4% of the sample.

Table 2 shows the weighted results of the logistic regression models. Odds ratios, standard errors, and *p*-values are presented. Consistent with previous studies [1,2], there was no significant sexual orientation difference in non-normal sleep duration across the different racial and ethnic groups with the exception of Latinx adults. The Latinx sexual minority adults were significantly more likely to report non-normal sleep duration than Latinx heterosexual adults. According to the NCHS, estimates are considered unreliable if the relative standard errors are equivalent to or higher than 30% [7]. The relative standard errors for all estimates predicting non-normal sleep duration were below 30% and were considered reliable.

Consistent with previous studies [1,2], sexual minority adults were significantly more likely to report not feeling rested than heterosexual adults across the different racial and ethnic groups, with the exception of Asian and Pacific Islander adults. There was no significant sexual orientation difference in not feeling rested among Asian and Pacific Islander adults. The relative standard errors for all estimates predicting not feeling rested were below 30% and were considered reliable.

Consistent with previous studies [1,2], sexual minority adults were significantly more likely to report having trouble falling asleep than heterosexual adults across the different racial and ethnic groups, with the exception of Asian and Pacific Islander adults. There was no significant sexual orientation difference in trouble falling asleep among Asian and Pacific Islander adults. The relative standard errors for all estimates predicting trouble falling asleep were below 30% and were considered reliable, with the exception of the estimate for Asian and Pacific Islander adults. The relative standard error for Asian and Pacific Islander adults was above 30%; accordingly, the odds ratio estimate for Asian and Pacific Islander adults was considered unreliable.

Consistent with previous studies [1,2], sexual minority adults were significantly more likely to report having trouble staying asleep than heterosexual adults across the different racial and ethnic groups. The relative standard errors for all estimates predicting trouble staying asleep were below 30% and were considered reliable. Finally, consistent with a previous study [2], sexual minority adults were significantly more likely to report taking medications for sleep than heterosexual adults across all racial and ethnic groups. The relative standard errors for all estimates predicting trouble falling asleep were below 30% and were considered reliable, with the exception of the estimate for Asian and Pacific Islander adults. The relative standard error for Asian and Pacific Islander adults was above 30%; accordingly, the odds ratio estimate for Asian and Pacific Islander adults was considered unreliable.

## 4. Discussion

Using five years of data from the National Health Interview Survey, this study examined whether there were significant sexual orientation differences in sleep problems in specific racial and ethnic populations. The analysis had a large sample size and enough statistical power to evaluate for any sexual orientation differences or non-differences in sleep problems in Latinx, non-Latinx Black, non-Latinx Asian and Pacific Islander, and non-Latinx White populations. Consistent with recent studies on sexual orientation and sleep problems, this analysis revealed that, for most racial and ethnic groups, there was no significant sexual orientation difference in non-normal sleep duration. Additionally, sexual minority adults were significantly more likely to report not feeling rested, trouble falling asleep, trouble staying asleep, and taking medications for sleep than heterosexual adults. 

There were two notable exceptions in the findings. The first exception was that Latinx sexual minority adults were significantly more likely to report non-normal sleep duration when compared with Latinx heterosexual adults. Chronic stressors, issues with acculturation, and ethnic discrimination are known to negatively affect sleep among Latinx adults [8]. In addition, psychological distress and BMI have been found to contribute to the association between ethnic discrimination and sleep problems among Latinx adults [9]. Studies should examine whether chronic stressors and health conditions affect Latinx sexual minority adults more than Latinx heterosexual adults, which may lead to a sexual orientation disparity in non-normal sleep duration. The second exception was that there was no significant sexual orientation difference in not feeling rested among Asian and Pacific Islander adults. A recent study found that many Asian and Pacific Islander sexual minority adults receive social support from family and friends [10]. Social support from family and friends should be examined regarding whether this has a positive and buffering effect for sleep quality among Asian and Pacific Islander sexual minority adults. 

Although this study has improved statistical power in comparison with past studies for producing estimates of sleep problems among specific racial and ethnic populations, there was not enough statistical power for several estimates in this study. Estimates for the associations between sexual orientation and the sleep problems of trouble falling asleep and taking medications for sleep among Asian and Pacific Islander adults were considered unreliable. An additional constraint of this study was not examining gender and sexual orientation identity (e.g., lesbian and gay versus bisexual) differences in the association between sexual orientation and sleep problems within each racial and ethnic group due to the lack of statistical power. Another constraint of this study, attributable to sample size limitations, was the lack of additional controls associated with sleep problems, such as depression, cardiometabolic health, and self-rated health. Another constraint attributable to the study sample size was the inability to analyze associations between sexual orientation and specific sleep durations, such as short sleep and long sleep problems. One study of Latinx adults found that ethnic discrimination increased the risk for short sleep and long sleep problems [8]. Latinx sexual minority adults may be more likely to experience short and long sleep problems than Latinx heterosexual adults. The availability of additional years of the NHIS data collection would create more opportunities to produce reliable estimates of sexual orientation differences or non-differences of sleep problems in specific racial and ethnic populations.

## Figures and Tables

**Table 1:** Weighted Percentages of Sexual Orientation Identity, Race and Ethnicity, and Sleep Problems, National Health Interview Survey, 2013–2017.

	Percentage
**Sexual Orientation Identity**	
Sexual Minority Status	2.7%
Heterosexual	95.9%
Missing	1.4%
**Race and Ethnicity**	
Latinx	13.1%
Non-Latinx Black	12.3%
Non-Latinx Asian and Pacific Islander	4.9%
Non-Latinx White	68.6%
Other Race and Ethnicity	1.1%
**Sleep Problems**	
Non-Normal Sleep Duration	37.5%
Not Feeling Rested	36.2%
Trouble Falling Asleep	15.7%
Trouble Staying Asleep	22.2%
Taking Medications For Sleep	8.4%

Note: *N* = 157,562.

**Table 2:** Weighted Odds Ratios and Standard Errors in the Associations Between Sexual Minority Status and Sleep Problems for Each Racial or Ethnic Group, National Health Interview Survey 2013–2017.

	Odds Ratio (SE)
**Sexual Minority Status Predicting**	
**Non-Normal Sleep Duration**	
Latinx	1.61 *** (0.17)
Non-Latinx Black	1.09 (0.11)
Non-Latinx Asian and Pacific Islander	1.25 (0.26)
Non-Latinx White	1.06 (0.05)
**Sexual Minority Status Predicting**	
**Not Feeling Rested**	
Latinx	1.28 * (0.14)
Non-Latinx Black	1.32 * (0.14)
Non-Latinx Asian and Pacific Islander	1.44 (0.28)
Non-Latinx White	1.23 *** (0.06)
**Sexual Minority Status Predicting**	
**Trouble Falling Asleep**	
Latinx	2.14 *** (0.26)
Non-Latinx Black	1.54 ** (0.19)
Non-Latinx Asian and Pacific Islander	1.37 (0.44)
Non-Latinx White	1.46 *** (0.09)
**Sexual Minority Status Predicting**	
**Trouble Staying Asleep**	
Latinx	1.82 *** (0.22)
Non-Latinx Black	1.43 ** (0.17)
Non-Latinx Asian and Pacific Islander	1.88 * (0.55)
Non-Latinx White	1.3 *** (0.07)
**Sexual Minority Status Predicting**	
**Taking Medications For Sleep**	
Latinx	2.95 *** (0.55)
Non-Latinx Black	2.32 *** (0.41)
Non-Latinx Asian and Pacific Islander	4.05 ** (2.03)
Non-Latinx White	1.98 *** (0.15)

Notes: SE = standard error. Models include controls (not shown) for missing information of sexual orientation identity, other race and ethnicity status, gender, age, education, and family poverty level. * *p* < 0.05, ** *p* < 0.01 *** *p* < 0.001. Two-tailed tests.
